# Genetic Algorithm-Based Trajectory Optimization for Digital Twin Robots

**DOI:** 10.3389/fbioe.2021.793782

**Published:** 2022-01-10

**Authors:** Xin Liu, Du Jiang, Bo Tao, Guozhang Jiang, Ying Sun, Jianyi Kong, Xiliang Tong, Guojun Zhao, Baojia Chen

**Affiliations:** ^1^ Key Laboratory of Metallurgical Equipment and Control Technology of Ministry of Education, Wuhan University of Science and Technology, Wuhan, China; ^2^ Research Center for Biomimetic Robot and Intelligent Measurement and Control, Wuhan University of Science and Technology, Wuhan, China; ^3^ Hubei Key Laboratory of Mechanical Transmission and Manufacturing Engineering, Wuhan University of Science and Technology, Wuhan, China; ^4^ Precision Manufacturing Research Institute, Wuhan University of Science and Technology, Wuhan, China; ^5^ Hubei Key Laboratory of Hydroelectric Machinery Design & Maintenance, Three Gorges University, Yichang, China

**Keywords:** genetic algorithm, mobile robot, digital twin, virtual model, trajectory optimization

## Abstract

Mobile robots have an important role in material handling in manufacturing and can be used for a variety of automated tasks. The accuracy of the robot’s moving trajectory has become a key issue affecting its work efficiency. This paper presents a method for optimizing the trajectory of the mobile robot based on the digital twin of the robot. The digital twin of the mobile robot is created by Unity, and the trajectory of the mobile robot is trained in the virtual environment and applied to the physical space. The simulation training in the virtual environment provides schemes for the actual movement of the robot. Based on the actual movement data returned by the physical robot, the preset trajectory of the virtual robot is dynamically adjusted, which in turn enables the correction of the movement trajectory of the physical robot. The contribution of this work is the use of genetic algorithms for path planning of robots, which enables trajectory optimization of mobile robots by reducing the error in the movement trajectory of physical robots through the interaction of virtual and real data. It provides a method to map learning in the virtual domain to the physical robot.

## 1 Introduction

Mobile robots are an important branch of the industrial robot family, and their demand accounts for about 30% of the demand for industrial robots (Niloy et al., 2021). Mobile robots integrate the comprehensive application technology of mechanical, computer, artificial intelligence and other disciplines, which can effectively improve the efficiency of industrial production, reduce the labor intensity of workers, and increase economic benefits (Duan et al., 2021). Mobile robots use sensors to sense the environment and achieve autonomous movement in complex environments according to rational algorithms (He et al., 2019; Chen et al., 2021a). Path planning is the planning of a collision-free path from the current position to the target position under the constraints using the relevant algorithms (Hu et al., 2019; Huang et al., 2021). Path planning algorithms include genetic algorithms, ant colony algorithms, etc. These algorithms are used in different situations due to different computational principles (Li et al., 2019a; Jiang et al., 2021a). Most of the current research on mobile robots uses improved algorithms to reduce their optimal path length or the number of iterations, and there is a lack of research on error control during the actual motion of the robot (Li et al., 2019b; Bai et al., 2021). In practice, the robot cannot follow the planned path to the target position during the movement due to the influence of environmental factors or the error in the coordination of various parts of the robot (Chen et al., 2021b; Chen et al., 2021c). At the same time, the lack of data feedback from most robots during movement makes it impossible to determine where errors occur and to correct the robot’s work path, making it impossible for the robot to work in workplaces with high operational accuracy (Yu et al., 2019; Luo et al., 2020).

With the introduction of national-level manufacturing development strategies such as the U.S. Industrial Internet, Germany’s Industry 4.0 and Made in China 2025, smart manufacturing has become a common trend and goal for global manufacturing development (Jiang et al., 2019a; Cheng et al., 2021). As a key technology to realize the concept and goal of smart manufacturing, digital twin has received wide attention from academia and is being applied in more and more industrial fields (Hao et al., 2021; Jiang et al., 2021b). Digital twin is a technical means to realize the information interaction between the physical world and the virtual world, which creates virtual models of physical entities digitally, simulates the operation of physical entities in the real environment with the help of actual data, and dynamically optimizes the working state of physical entities (Tao et al., 2019; Lu et al., 2020; Jiang et al., 2021a). As a technology that makes full use of models and data and integrates multiple disciplines, the digital twin is oriented to the whole product lifecycle process, playing the role of a bridge and link between the physical world and the information world to provide more real-time, efficient and intelligent services (Jiang et al., 2019c; Huang et al., 2020; Liao et al., 2021). Digital twin-based precision distribution for production logistics is a combined application of digital twin technology and mobile robots (Li et al., 2019c; Li et al., 2020). Production logistics, including internal logistics and external logistics, is the key to ensure normal production, improve production efficiency and reduce product costs (Liu J et al., 2021; Yang et al., 2021). Digital twin production logistics refers to a new production logistics operation mode driven by twin data, through real mapping of physical entities and virtual models, real-time interaction and closed-loop control, to achieve task combination optimization, transportation path planning and transportation process control of production logistics, so as to achieve seamless and intelligent production process logistics (Sun et al., 2020b; Liu et al., 2021b).

In this paper, we develop a mobile robot based on digital twin technology, build a virtual environment in Unity, and complete the path planning of the virtual model using genetic algorithm. A communication architecture between the virtual model and the physical robot is proposed to complete the data interaction between the robot and the virtual model via Bluetooth, which achieves dynamic optimization of the physical robot movement process and improve the accuracy of the robot’s movement trajectory. In contrast to the work done by others, the focus of our work is to emphasize the real-time mastery and correction of errors generated during robot movement, and to achieve the reduction of errors in physical entities through the interaction of virtual models and physical entities. There are three contributions of this paper.

1) A robot that can move autonomously was developed.
2) A digital twin of the mobile robot was built in Unity.
3) The path planning of the robot was implemented in a virtual environment based on genetic algorithm.
4) A trajectory optimization method for a mobile robot was proposed. The trajectory error of the robot is gradually reduced by virtual-real interaction.

The rest of this paper is organized as follows. Reviewed some of the research done by domestic and international scholars on mobile robots and digital twins in Section 2. Section 3 describes the virtual environment of the robot and the genetic algorithm-based path planning in the virtual environment. Section 4 presents the experiments of digital twin-based mobile robot trajectory optimization and summarizes the experimental results. Section 5 concludes the paper with summary and future research directions.

## 2 Related Work

Mobile robots have the characteristics of high efficiency, wide working range and convenient operation, etc. With the continuous enrichment of computer control theory and deep learning and other related theories, the requirements for the movement accuracy of robots have gradually increased Material and Methods (Huang et al., 2019; Miao et al., 2021). Robots with high motion accuracy can improve efficiency and avoid wasting resources when moving with high accuracy requirements, especially when completing transportation tasks in high-risk production areas (Ma et al., 2020; Sun et al., 2020a; Liu et al., 2021a). HUR Sung Wook et al. propose a new approach to efficient trajectory optimization that exploits the fact that the dynamics of a deterministic system is uniquely determined by the initial state and control over the time horizon of interest (Hur et al., 2021). Hu proposed a method based on content image retrieval to identify obstacles on the robot’s running path (Hu, 2021). Fu et al. analyzed the local minima problem and the target unreachability problem which are easy to occur in the artificial potential field method, and solved the local minima problem of the artificial potential field method better by introducing the virtual obstacle model (Fu et al., 2021). Huo et al. proposed an optimal fuzzy logic obstacle avoidance algorithm for implementing motion obstacle avoidance of mobile robots (Huo and Wang 2021). Zheng et al. proposed a laser-based person detection and obstacle avoidance algorithm for differential drive robots applied to a handling robot to transport materials along a reference path in the hospital field (Zheng et al., 2021). Luka Petrović et al. proposed a new trajectory planning algorithm using stochastic optimization in order to find a continuous-time Gaussian process for collision-free trajectory generation (Petrović et al., 2020). Deng et al. proposed a multi-obstacle path planning and optimization method that uses convex packages to optimize the base obstacles and obtain the corresponding set of base obstacle points, and uses cubic bezier curves to smooth the path to fit the kinematic model of the robot (Deng et al., 2021).

The concept of digital twin was proposed by Prof. Michael Grieves, who showed in his paper that virtual models and related subsystems are constructed to represent physical entities in a virtual information space and establish two-way dynamic connections through data from physical devices in real space, but due to technical limitations at that time, the concept of digital twin did not gain much attention (Tan et al., 2020; Sun et al., 2021). In 2011, NASA applied the digital twin concept to the Apollo project to construct virtual bodies of space vehicles in virtual information space, and through the observation and analysis of the virtual bodies, the prediction and maintenance of the flight status of space vehicles were realized, and then the digital twin technology began to attract attention (Tao et al., 2018; Liu Y et al., 2021a). Many internationally renowned companies have already started to explore the application of digital twin technology in product design, manufacturing and service (Sun et al., 2020c; Tao et al., 2021). In product design, for the innovative design of complex products, Dassault has established a 3D experience platform based on the digital twin, which uses the information from user interaction to continuously improve the product design model in the information world and to implement it into the physical product improvement (Sun et al., 2020d; Fu et al., 2021). In manufacturing, Siemens has built a production system model that integrates manufacturing processes based on the digital twin concept, formed a virtual enterprise based on the model and an enterprise mirror based on automation technology, and carried out application validation in the production process of Siemens industrial equipment: Nanobox PC. In terms of product service, PTC has made digital twin a key aspect of intelligent and connected products and is committed to establishing a real-time connection between the virtual world and the real world, enabling predictive maintenance of products and providing customers with efficient product after-sales service and support.

The digital twin technology mainly includes important parts such as the creation of virtual models, the collection of actual data, and the data interaction between physical entities and virtual models. The determination of model parameters, the fit constraints between components and the accuracy of the model are the key issues and difficult problems of the virtual model. Currently, research has been conducted in the framework and process of digital twin modeling, and there are many software for modeling, but there is a lack of a complete set of modeling theory and modeling process (Jones et al., 2020; Liu et al., 2020). Digital twin technology requires a high level of real-time data, and in most occasions automated data collection is used, which relies on the use of devices with good communication conditions and various types of sensors. Data interaction between physical entities and virtual models is the key to the implementation of digital twin technology, but there is little research related to the interaction and collaboration between machines and services (Zhuang et al., 2017; Guo et al., 2021; Ruzsa, 2021). Verner et al. developed a system of reinforcement learning scenarios in which humanoid robots learn the protocols required to lift weights of unknown mass by exploring state space. To speed up the process of physical training, these experiments were performed in a virtual space, simulated in a digital twin, where the parameters obtained from simulation learning were mapped onto the physical robot (Verner et al., 2018). Huang et al. demonstrated a linear tracking robot trained in digital twin mode in a virtual space (Huang et al., 2019). Matulis et al. present a method for creating and training a digital twin robot for a robotic arm. The project demonstrates that a trained robot can perform a given task even if it is currently in a state it has never been in before (Matulis and Harvey, 2021). Liu et al. proposed a digital-driven machining quality tracking and dynamic control method, which effectively solved the problems of low efficiency of quality problem traceability, poor timeliness and unpredictability of quality control in machining process (Liu Y et al., 2021b).

The application of digital twin technology in logistics and distribution is one of the future directions of digital twin, which realizes the accurate distribution of goods through the control of the distribution process of mobile robots. Currently, most of the domestic research on mobile robots considers the robot’s movement path planning. From the overall effect, the improved path planning algorithm improves the movement accuracy of the robot, but it is impossible to know the location where the movement trajectory error occurs and lacks feedback on the error during the robot movement (Liu et al., 2021d). Applying digital twin technology to mobile robots and establishing the interaction between robots and virtual models can grasp the robot’s operation in real time and optimize the robot’s movement trajectory (Zhao et al., 2021). Digital twin-based mobile robots can be applied to applications where trajectory attractions are demanding, such as power station inspection, space exploration, etc.

## 3 Materials and Methods

**FIGURE 1:**
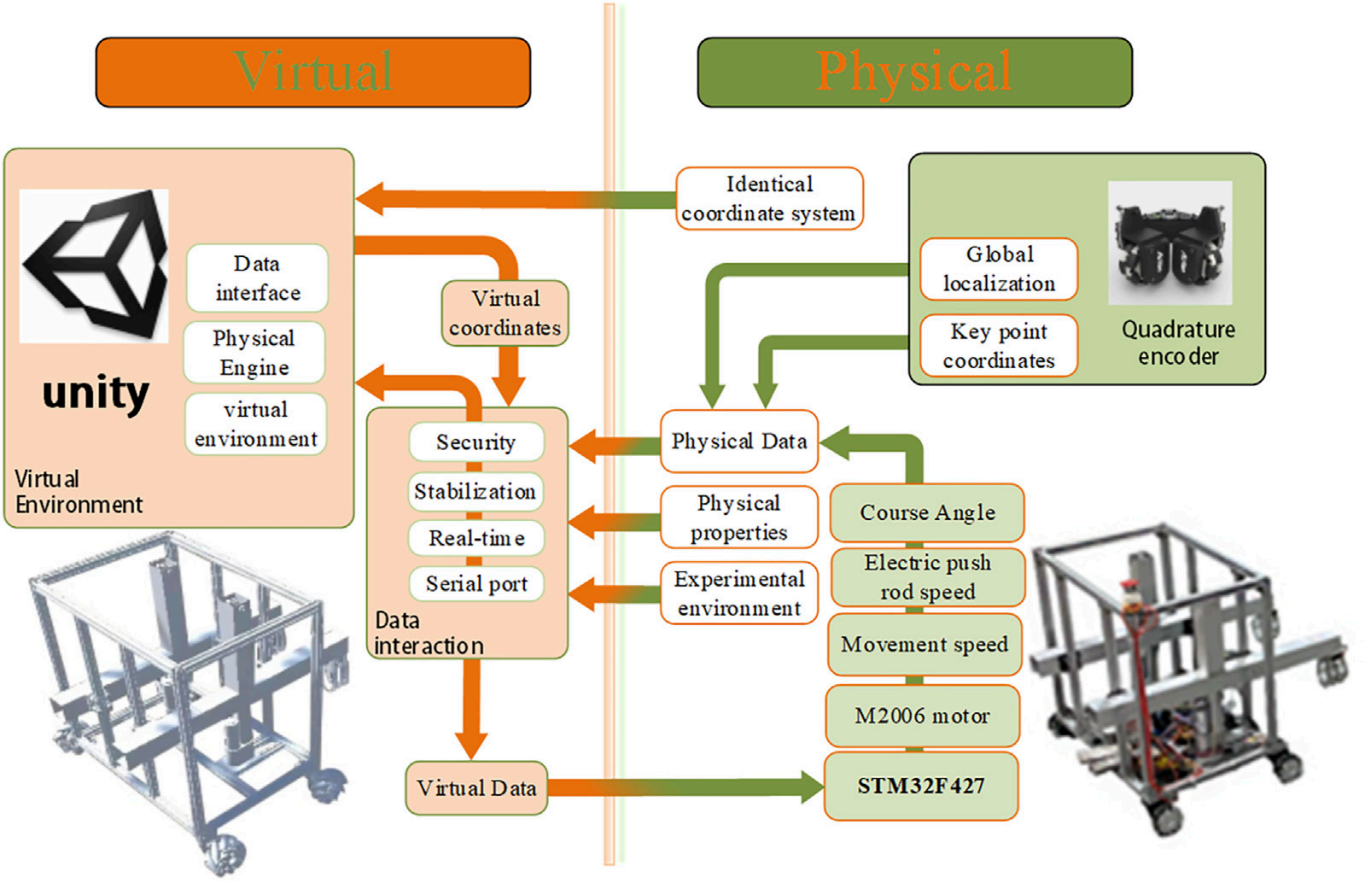
Framework for interaction between virtual models and physical entities.

This section describes the general approach and workflow for improving the trajectory accuracy of mobile robots, including the setup of virtual environments and genetic algorithm-based path planning. Figure 1 provides a functional overview of the digital twin robot virtual-reality interaction method. The robot entity is controlled by STM32 microcontroller, the orthogonal code disk determines the spatial coordinates of the robot and creates a virtual model of the robot in untiy. STM32 establishes communication with Unity’s virtual serial port to achieve data interaction between the physical entity and the virtual model.

### 3.1 Physical Robot

**FIGURE 2:**
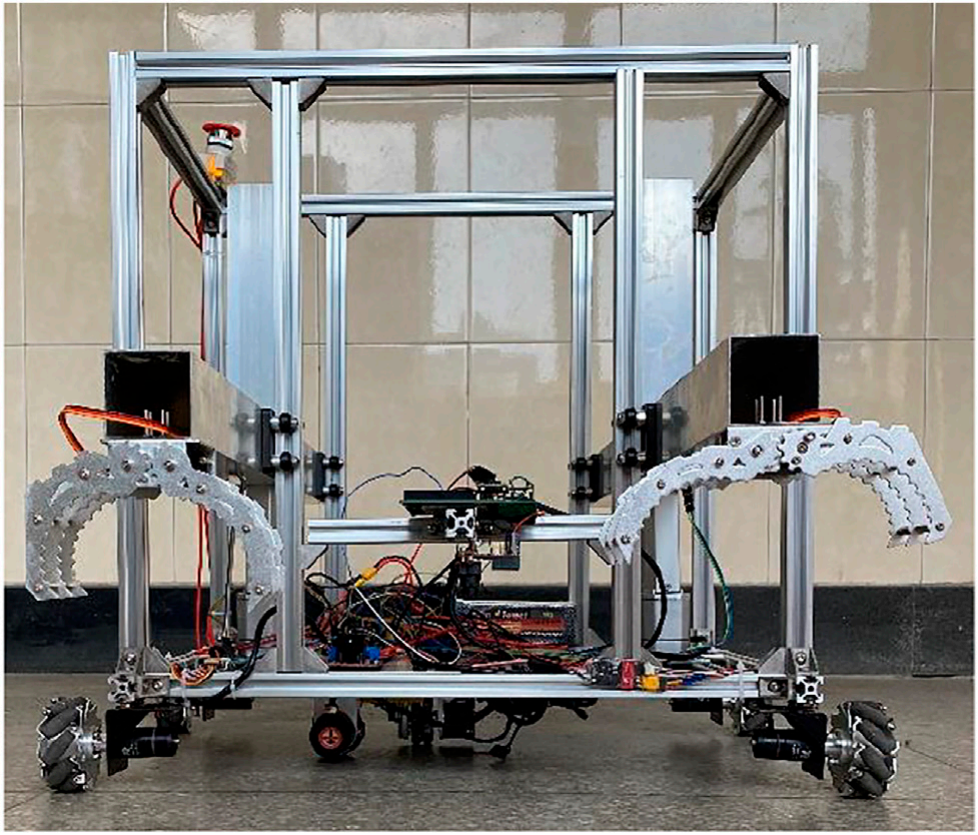
Physical structure of the robot.

The physical entity is objective and usually consists of control subsystems, power subsystems, actuation subsystems, etc. and accomplishes specific tasks through collaboration among the subsystems, and its environmental data and operational status are monitored in real time by sensors deployed on the physical entity (Weng et al., 2021). The robot is built from aluminum profiles and driven by RoboMaster M2006 DC brushless motors. The robot weighs about 13 kg and can achieve movements such as straight line movement, lateral movement, turning and other movements. The motors provide a maximum speed of up to 500 rpm, a maximum sustained torque of 1,000 mN m, and a maximum sustained output power of 44 W. Each motor is controlled by a 32-bit microprocessor STM32 board. The STM32 board controls the M2006 motor to drive the robot movement via the c610 electronic speed controller, using an orthogonal encoder and gyroscope to determine the robot’s position coordinates. The orthogonal encoder has two directions, X and Y, corresponding to one coordinate on the plane. By giving the coordinates, it can realize the fixed-point movement of the robot, and its combination with the gyroscope can realize the correction of the running trajectory by controlling the number of revolutions of the motors. The L298N module controls the electric actuators and mechanical jaws to grasp the object. The physical entity of the robot is shown in Figure 2.

### 3.2 Virtual Environment Construction

The virtual model is a digital mirror of the physical entity and mainly includes four layers of models: geometry, behavior, interaction, and association. Geometric models mainly describe geometric relationships such as size and shape (Xiao et al., 2021). Behavioral models analyze expected behavior, actual behavior, and random behavior. Interaction model refers to the data interaction, behavior interaction, information interaction between virtual model and physical entity (Liu Y et al., 2021c; Yun et al., 2021). The association model describes the interactions between the geometric model, the behavioral model, and the interaction model.

**FIGURE 3:**
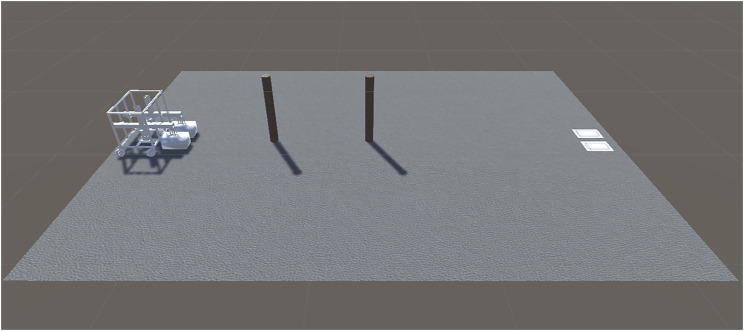
Virtual environment.

Unity was chosen to complete the construction of the virtual model, the motion control of the model, and the development of the experimental scenes. In Unity, a robot movement scene is built as shown in Figure 3, which includes a virtual model of the robot, two cylindrical obstacles, a white goods stacking area, and handing objects. When constructing the virtual model, it is necessary to consider the influence of realistic factors, such as the robot’s material, mass, and the robot’s movement speed. The specific parameters of the experimental environment are shown in Table 1. The requirement for the robot’s movement path is that the robot starts from the starting position, carries the handling objects through two obstacles, and finally reaches the white goods stacking area. The ability of the robot to place the load in the stacking area is a criterion for determining whether the robot is experiencing trajectory errors. There is a maximum speed limit on the movement of the physical robot and a height limit on the rise of the electric actuators, which must be captured and applied to the virtual environment during the experiment.

**TABLE 1:** Experimental environment parameters.

Facility name	Dimensional parameters
Grey base plate	3 m × 5 m
Obstacles	d = 0.1 m; h = 1 m
Stacking area	0.4 m × 0.3 m
Handling objects	0.28 m × 0.16 m × 0.15 m
Distance between obstacles	1.2 m

### 3.3 Genetic Algorithm-Based Path Planning

**FIGURE 4:**
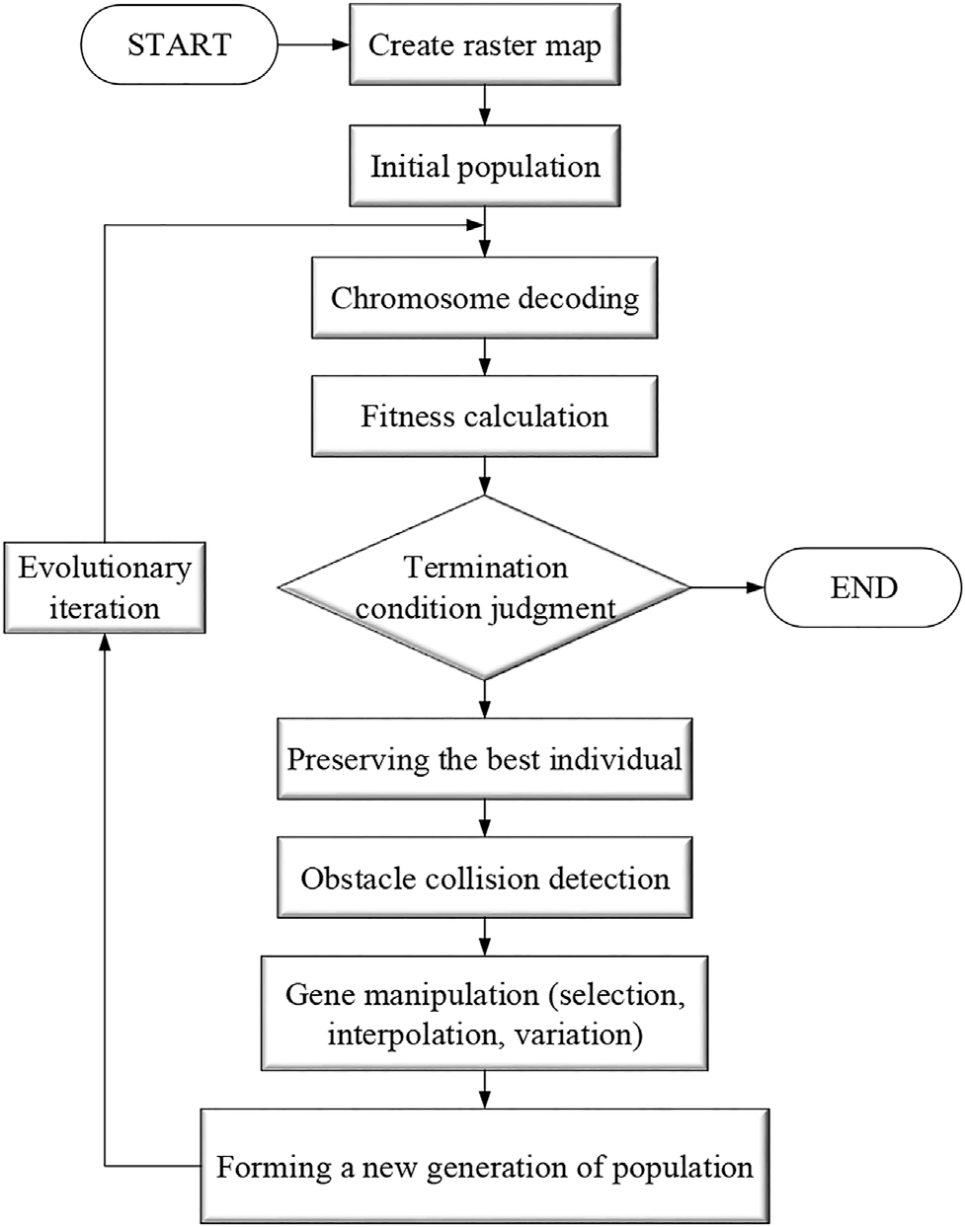
Genetic algorithm flow chart.

Genetic algorithms are stochastic global search optimization methods that simulate the phenomena of replication, crossover, and variation that occur in natural selection and inheritance. Starting from an initial population, the population evolves to increasingly better regions in the search space by random selection, crossover and mutation operations to produce a group of individuals better suited to the environment, and finally converges to a group of individuals best suited to the environment to obtain a quality solution to the problem (Nazarahari et al., 2019; Sarkar et al., 2020). For path planning based on genetic algorithm, the individuals suitable for the environment are the suitable moving paths, and the one that best satisfies the conditions is obtained by random selection and crossover variation. The flow chart of virtual environment robot path planning based on genetic algorithm is shown in Figure 4.

The steps of genetic algorithm-based path planning for virtual environment robots are as follows.

**FIGURE 5:**
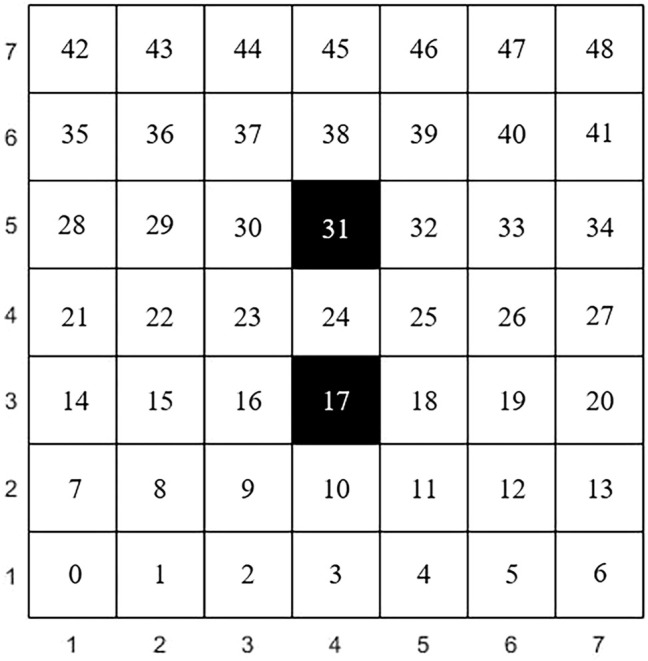
Raster map.

1) The raster method is used to model the robot’s walking space, which is represented by a square. The white grid indicates the moveable area and the black grid indicates the obstacle. In this paper, the robot walking space is shown in Figure 5.

The Cartesian coordinate system is established with the first grid in the lower left corner of the map as the coordinate origin, so the coordinates of each grid can be expressed as (x, y). For example, the first grid in the bottom left corner can be represented as (1,1). The number in the raster represents the number *n*. The conversion formula between number and coordinate is shown in Equation 1. In this paper, the grid numbers of the starting and ending points are 3 and 45, and the grid numbers of the obstacles are 17 and 31, respectively.

$$\left\{ \begin{array}{l} x=\mathrm{int}\left(n/7\right)+1\\ y=n\%7+1 \end{array}\right.$$ (1)

2) Initialize chromosomes and use floating point encoding to form populations. The purpose of initializing the population is to randomly generate multiple feasible paths, feasible paths being those that do not collide with the obstacle grid.
3) To allow the dominant individual to be preserved, define the fitness function as shown in Equation 2.

$$f_{fitvalue}=\exp\left(200/dis*\mu \right)-1$$ (2)

Where dis is the distance of each individual (path), μ is the collision coefficient between the individual and the obstacles. If the path collides with the obstacle, then μ take 0, if no collision then μ take 1. Obviously, if the path collides with an obstacle, its fitness is 0. It will not be inherited to the next generation, and this individual is discarded. dis is calculated using the Euclidean distance, and the calculation formula is shown in Equation 3.

$$dis_{i+1}=\sqrt{{\left(x_{i+1}-x_{i}\right)}^{2}+{\left(y_{i+1}-y_{i}\right)}^{2}}$$ (3)

4) A number of individuals were selected from the population using the roulette selection method. In this method, the selection probability of each individual is proportional to its fitness value, and the higher the fitness value, the higher the probability of being selected. The specific steps are as follows.
1) Calculate the fitness of each individual in the population.
2) Calculate the probability of each individual being inherited into the next generation population, as shown in Equation 4.

$$P\left(dis_{i}\right)=\frac{f_{fitvalue}\left(dis_{i}\right)}{\sum_{j=1}^{N}f_{fitvalue}\left(dis_{i}\right)}$$ (4)

3) Calculate the cumulative probability of each individual, as shown in Equation 5.

$$q_{i}=\sum_{j=1}^{i}P\left(dis_{i}\right)$$ (5)

4) Generate a uniformly distributed pseudo-random number r in the interval [0,1]. If *q*_*k*-1_< r ≤ *q*_*k*_ is satisfied, select individual k .
5) A random number approach is chosen for chromosome crossover operations to form new chromosomes, as shown in Equation 6. where *a* is the generated random number, *s*_*i*_ is the child, $fat_{i}$ and $fat_{i-1}$ are the parents.

$$s_{i}=\left(1-a\right)*fat_{i}+a*fat_{i-1}$$ (6)

6) The uniform variation operator is selected for variation operations. The original gene values at each locus in the individual coding string are replaced with a random number that matches a uniform distribution within a certain range with a certain small probability.
7) Formation of new individuals of the next generation. Calculate the fitness value of the new individual.
8) Determine whether the best individuals in the new generation population meet the expected requirements, and if so, output the result and go to step (I), otherwise, return to step (E).
9) Define two termination criteria: 1) Maximum number of iterations is 100; 2) Little chromosome variation and population stabilization. Before each genetic operation, determine whether the termination criterion is satisfied, and if the condition is satisfied, the optimization process ends. The specific parameters are shown in Table 2.

**TABLE 2:** Genetic algorithm parameter settings.

Parameters	Value
Population size	50
Evolutionary algebra	100
Number of chromosomes	5
Mutation probability	0.045
Gene conversion probability	0.1
Gene crossover probability	0.9
Gene variation probability	0.07
Select Strategy	Roulette

**FIGURE 6:**
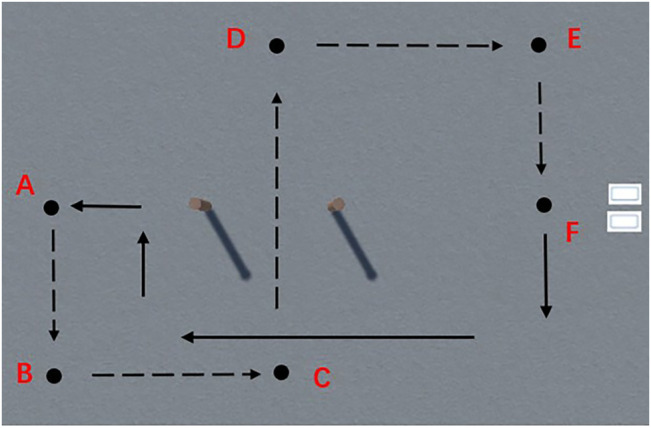
Virtual robot movement path.

According to the decoding method of genetic algorithm, the input is the coordinates of the starting point, target point and obstacles, and the output is the corresponding motion trajectory. According to the actual working conditions and task requirements of the physical robot, the motion trajectory of the robot is determined as shown in Figure 6. point A and point F denote the starting point and end point respectively, and points B, C, D and E are path critical points. The physical robot follows the dashed path from the starting point A through the obstacles to the end point F, and then returns along the solid path.

## 4 Results

### 4.1 Experimental Procedure

In digital twin technology, the virtual model serves as a realistic mapping, simulation, and feedback correction. After several simulation tests in the virtual environment, appropriate operating parameters are determined and sent to the physical entity to realize the control of the virtual model over the physical entity (Liu J et al., 2021). Before the experiment starts, set the moving speed of the physical robot and the lifting speed of the electric actuator to constant values. Since the physical robot uses an orthogonal encoder for global positioning, the directional movement of the physical robot can be achieved by specifying the location coordinates of the target point.

**FIGURE 7:**
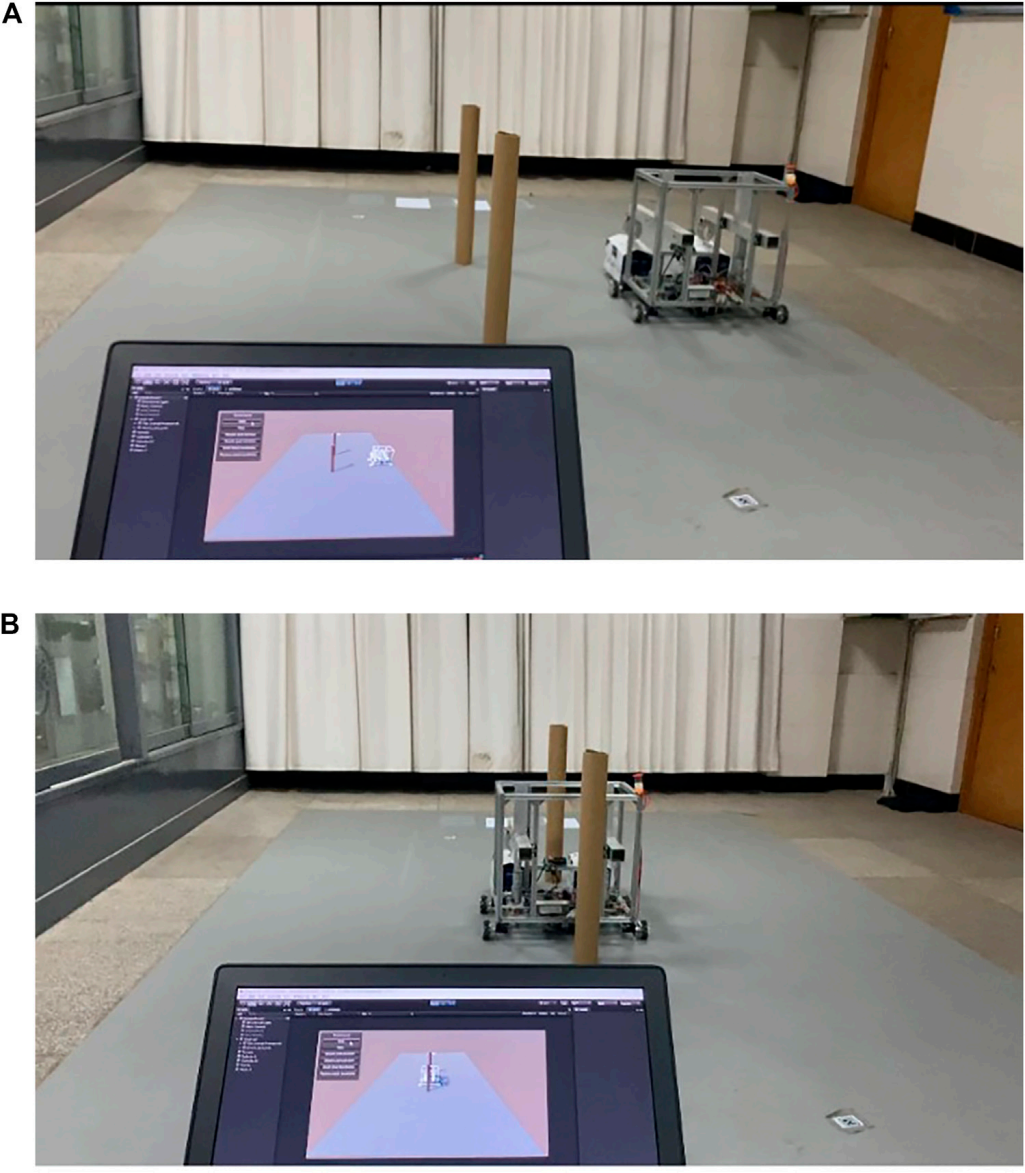
Virtual models control the movement of physical robots. Panel **(A)** indicates that the robot passes the path point C; Panel **(B)** shows the robot passes an obstacle.

The specific operation process is: establish the same global coordinate system as the virtual environment in the physical robot control system, send the coordinates of points A, B, C, D, E and F in Figure 5 to the physical robot STM32 board, then the robot can move according to the coordinates after receiving the path coordinates. The movement process of the robot is shown in Figure 7. From the figure, it is clear that the physical robot moves according to the path obtained by training in the virtual environment.

**FIGURE 8:**
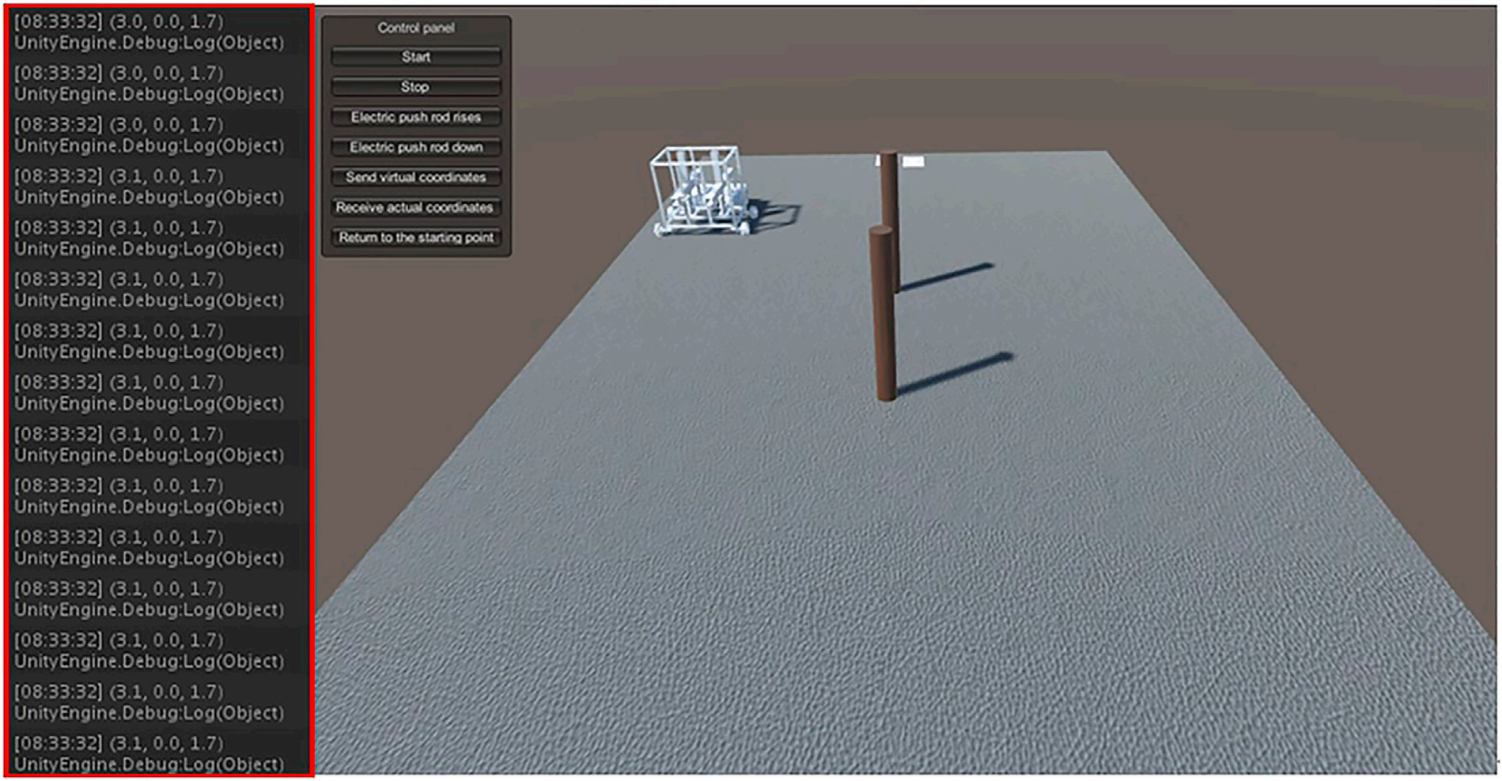
Virtual environments receive real-time movement data from physical robot.

After the physical robot starts moving, the STM32 board sends the real-time coordinates of the moving path to the virtual environment. The virtual model moves in the virtual environment based on the obtained real-time coordinates, and determines the position of the robot trajectory offset by comparing it with the predefined running trajectory. The coordinates of the key points of the path are continuously adjusted according to the trajectory offset, thus gradually improving the accuracy of the actual moving trajectory of the physical robot. The experimental procedure is shown in Figure 8, where the red rectangular box shows the real-time data of the physical robot movement received by the virtual environment.

### 4.2 Analysis of Errors and Sensitivities

The physical robot cannot reach the position of the specified coordinate point during the movement. The reasons for this phenomenon are measurement error, physical error, virtual error and other aspects. Measurement error refers to the dimensional measurement error and assembly error that occurs during the building process of the physical robot. Physical errors are the errors between the robot and the environment and the errors in the robot hardware, mainly the friction between the ground and the wheels, the encoder error and the gyroscope heading angle error. Virtual errors refer to the model dimensional errors and virtual model assembly errors that occur when modeling in the virtual environment.

Sensitivity analysis is the process of finding out the most influential factor on the experimental results among many uncertainties and analyzing the degree of its influence on the experimental target. Among several types of errors affecting the accuracy of robot motion trajectory, both measurement errors and virtual errors can be artificially controlled to reduce the occurrence of errors. Physical errors are random in nature and uncontrollable. Considering several factors that generate physical errors, among them, the friction between the ground and the wheels has the greatest influence on the experimental results, so the influence of other errors is ignored and only the influence brought by the friction between the ground and the wheels is considered.

### Analysis of Experimental Results

**FIGURE 9:**
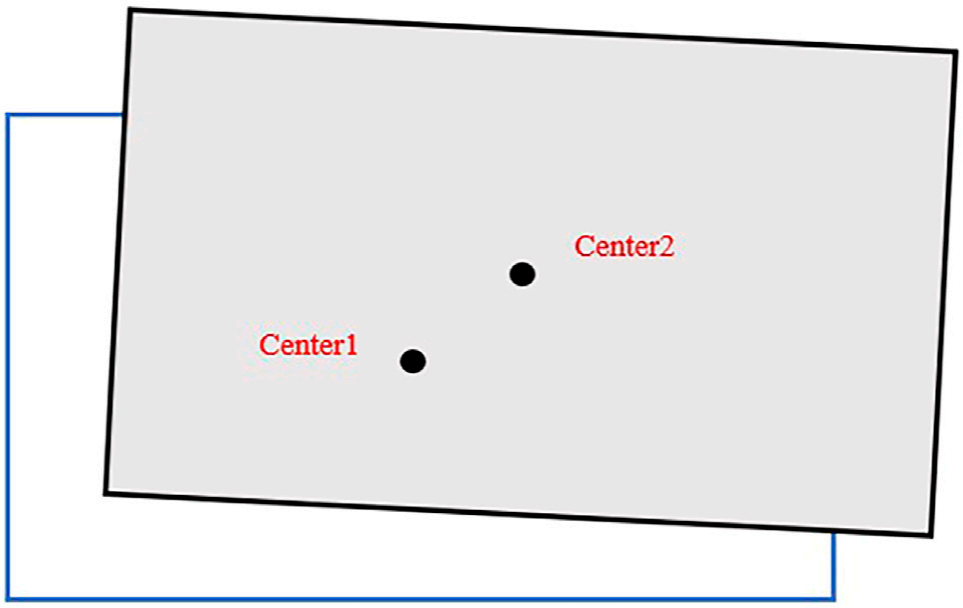
Deviations in cargo placement.

The reason for setting the stacking area is that it is difficult to observe whether the trajectory deviation occurs during the actual movement of the robot, and it can be judged whether the robot shifts during the movement by whether the robot can place the goods correctly in the stacking area. Figure 9 shows a situation where the goods are not placed correctly, from which it can be determined that the physical robot has shifted during the movement. The larger the area of cargo deviation from the stacking area, the larger the amount of robot trajectory deviation.

**FIGURE 10:**
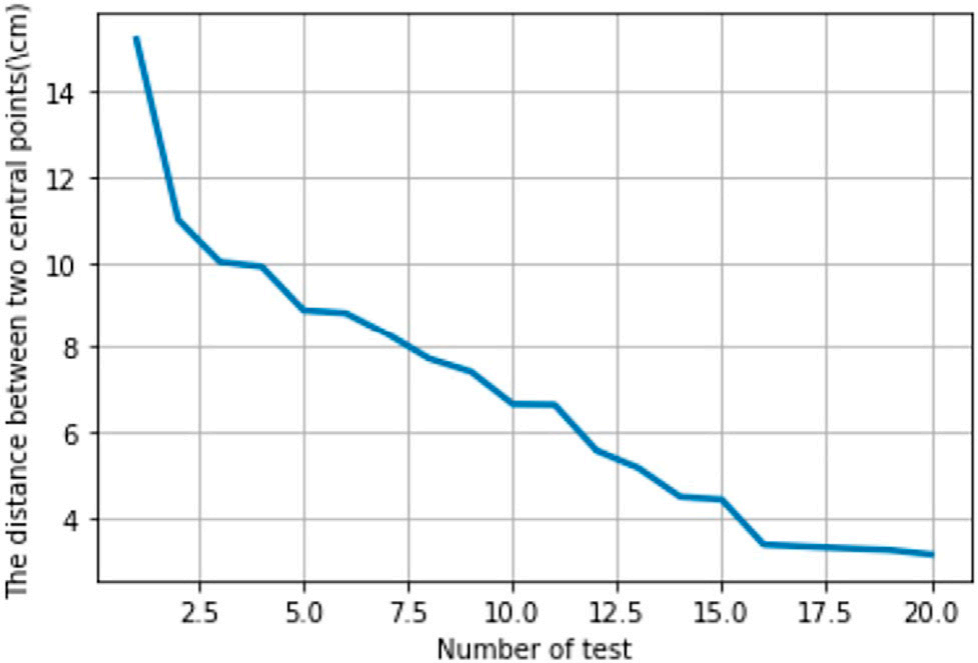
Trend of distance between two center points.

Center1 is used to indicate the center of the bottom surface of the goods, and Center2 indicates the center of the stacking area. The distance between Center1 and Center2 is used to indicate the degree of deviation of the goods not correctly placed in the stacking area, as shown in Figure 8. The experimental procedure in Section 4.1 is repeated 20 times to obtain the graph of the variation of the distance between the two center points, as shown in Figure 10. As can be seen from Figure 10, the distance between the two center points gradually decreases with the increase of the number of experiments, which laterally reflects that the offset of the robot’s moving trajectory is gradually decreasing, thus improving the robot’s moving accuracy and realizing the optimization of the robot’s moving trajectory.

**FIGURE 11:**
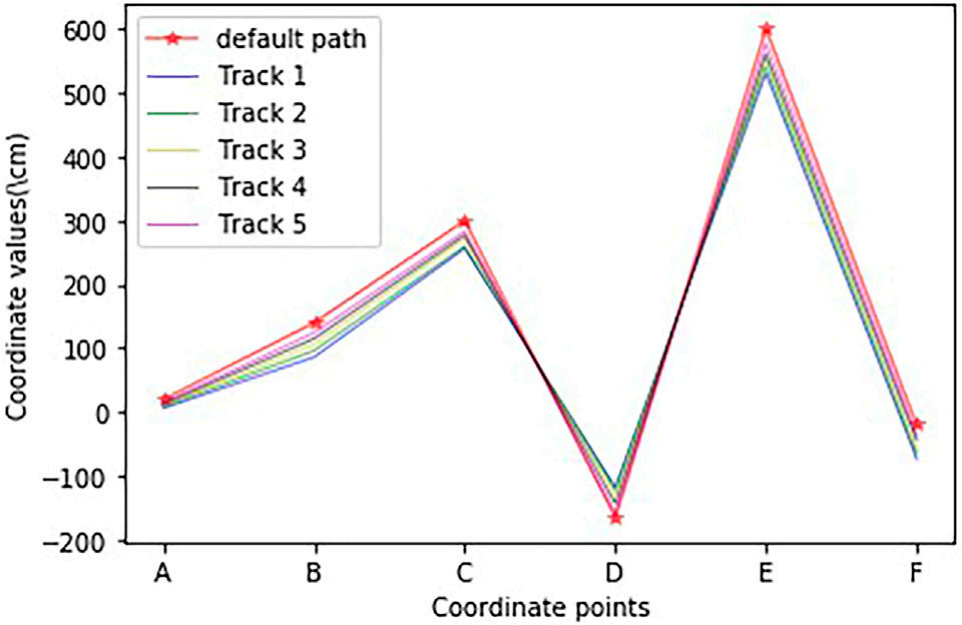
Comparison of coordinate changes of the first five movement trajectories.

Further, to determine the location where the robot’s trajectory deviation occurred, the first five experimental paths were selected, and the actual coordinates of six key points were recorded and compared with the preset key point coordinates. The results are shown in Figure 11, and the red pentagons are the coordinates of the preset path key points. From the overall change, the actual moving path gradually approximates the preset path as the number of experiments increases; In terms of local variation, the trajectory coordinates of points B, D, and E vary greatly. After analysis, the reason for this phenomenon may be that the friction force on the robot becomes larger during the lateral translation of the robot, resulting in the robot not reaching the preset key point.

## 5 Discussion

In this paper, the trajectory optimization of mobile robots is achieved through digital twin technology. A mobile robot was designed, using the STM32 to control the movement of the robot and to receive data. A virtual model corresponding to a physical entity as well as a virtual environment that is the same as the real scene are created in Unity. Based on the data interaction characteristics of the digital twin technology, the robot’s movement path is dynamically adjusted, the robot’s movement accuracy is improved, and the closed-loop control combining virtual and actual is realized. The purpose of this work is not to innovate path planning algorithms, but to apply digital twin technology to mobile robots as an applied innovation. In contrast to the work done by others, this work focuses on the establishment of a digital twin of the mobile robot, the completion of the communication between the virtual model and the physical entity, and the realization of the trajectory optimization of the mobile robot.

This paper provides a reference for the application of digital twin technology in logistics and transportation industry, and provides a method to solve the connection between virtual models and physical entities, which is beneficial to promote the application of digital twin in other industries. However, the movement path of the robot in this paper is relatively simple and there are fewer obstacles on the movement path. In future work, it is necessary to consider how to improve the trajectory accuracy of the robot under more complex moving paths and to consider the time complexity and computational complexity of the algorithm. Meanwhile, the construction method of virtual model and the real time of real and virtual data interaction also need to be further studied.

## Data Availability

The raw data supporting the conclusion of this article will be made available by the authors, without undue reservation.
